# Effects of a 12-Week Pilates Program on Functional Physical Fitness and Basal Metabolic Rate in Community-Dwelling Middle-Aged Women: A Quasi-Experimental Study

**DOI:** 10.3390/ijerph192316157

**Published:** 2022-12-02

**Authors:** Chien-Hsiao Su, Hsuen-Ying Peng, Cheng-Wen Tien, Wen-Ching Huang

**Affiliations:** 1 Department of Exercise and Health Science, National Taipei University of Nursing and Health Sciences, Taipei 112303, Taiwan; 2 Department of Rehabilitation, Camillian St. Mary’s Hospital Luodong, Yilan 265502, Taiwan; 3 Physical Education Office, General Education Center, National Taipei University of Nursing and Health Sciences, Taipei 112303, Taiwan

**Keywords:** community, fall prevention, functional fitness, health promotion, neuromotor exercise, Pilates

## Abstract

Background: The aging society worldwide carries public and inevitable issues. Aging is accompanied by multiple diseases, and the health impacts challenge healthcare and social systems. In addition to medical treatment, exercise has been recognized as an effective strategy not only for disease prevention and alleviation, but also for multiple health benefits on health promotion. The purpose of this study was to investigate the effects of a suitable Pilates exercise intervention program on health maintenance and benefits in community-dwelling middle-aged women with a quasi-experimental design. Methods: We recruited healthy middle-aged community-dwelling women who had not regularly exercised in the previous three months. The participants were assigned to the experimental (*n* = 22) and control (*n* = 23) groups based on a quasi-experimental design. The experimental group participated in a mat-based Pilates exercise class twice a week (1 h/session) throughout the 12-week intervention, whereas there was no intervention for the control group. Body composition, basal metabolic rate, and functional physical fitness—comprising cardiovascular capacity, flexibility, muscular strength of upper limbs, muscular strength of lower limbs, core strength, agility, static balance, and dynamic balance—were assessed as primary outcomes in both groups before and after the intervention. Results: There were no significant differences in any of the dependent variables between the two groups before the exercise intervention. After the 12-week intervention, body composition, including body mass index, body fat (−1.5 to 3%), and basal metabolic rate (+10.6%), and functional fitness, including flexibility (+3.5%), core strength (+31.5%), lower-limb strength (+13.5%), agility (+7.3%), and balance (+4.2%), improved significantly in the experimental group relative to the control group (*p* < 0.05). Moreover, the improvement in physical fitness in lower-limb strength, agility, and balance for fall prevention also demonstrated higher clinical significance than the control. Conclusions: This 12-week mat-based Pilates exercise program significantly improved body composition, basal metabolic rate, and functional physical fitness in community-dwelling middle-aged women. The beneficial effects of Pilates exercise programs may thus promote improved health in the middle-aged female population, with practical implications for communities.

## 1. Introduction

Pilates, which was originally called Contrology, is a type of whole-body exercise designed to improve daily activity and develop functional and sustainable body movement patterns with low impact. The principle of Pilates is to use centering, concentration, control, breath, precision, and fluidity to achieve a mechanical advantage that results in optimal balance, strength, and health [1]. Pilates exercise training should be considered as a prescribed exercise for the older population, since it improves fall prevention, physical fitness, and mood states [2], and also demonstrates greater effects on balance abilities for the risk of falls in the elderly [3,4]. Quality of life, depression, and anxiety can be also effectively improved by Pilates and aerobic exercise in overweight/obese individuals [5], and the body weight, BMI, and body fat percentage in adults who are overweight or obese could be dramatically reduced in meta-analysis [6]. Furthermore, Pilates can be used as an alternative therapeutic option for chronic nonspecific lower back pain, with specific programs and training frequencies [7]. In older adults with amelioration of chronic lower back pain, Pilates showed more effective effects compared to aerobic exercise because the trunk stabilization muscles were targeted [8]. Pilates also has beneficial effects for pregnancy and labor, in that it results in improvements in grip strength, hamstring flexibility, spinal curvature, labor pain intensity, and the labor process [9,10]. Pilates-based exercise can thus have multiple beneficial effects and can be used for general health promotion and as a therapeutic strategy in various populations.

The proportion of the world’s population that is aged over 60-years-old will almost double, from 12% to 22%, between 2015 and 2050 [11]. Therefore, all governments will have to address various associated challenges to ensure that their health and social systems can accommodate this demographic shift. An aging society has critical impacts not only on the economy, but also on the health system. The mechanisms causing age-related diseases such as Alzheimer’s disease, cancer, maculopathy, osteoporosis, rheumatoid arthritis, and sarcopenia are similar to those associated with the aging process, such as inflammation, oxidation, cellular senescence, and changes in the gut microbiota [12]. With respect to gender differences, estrogen deficiency in middle-aged women during the menopause and perimenopause stages is associated with the above diseases. Estrogen deficiency may be a major pathogenic element that disrupts energy homeostasis, resulting in the development of obesity through hypothalamic inflammation [13]. It may also contribute to an increased incidence of sarcopenia by inhibiting muscular satellite cell proliferation [14]. Estrogen deficiency-related primary osteoporosis and fractures are also becoming important public health concerns for middle-aged women [15].

The beneficial effects of exercise interventions such as tai chi, high-intensity aerobic exercise, eight-section brocade exercise, Pilates, and closed kinetic chain exercise on postmenopausal osteoporosis were recently explored in a systematic review [16]. According to a community study, lower-limb muscle strength and trunk flexibility were significantly improved in adult women who had participated in a Pilates intervention [17]. Pilates exercise programs may also improve the physical fitness of middle-aged women because they can be adjusted according to the participants’ age, level of fitness, and specific physiological conditions [18]. Motor performance and obesity indices in older women with obesity can also be improved by Pilates exercise programs [19]. In addition, falls are also considered a primary cause of morbidity and disability in the older population, and physical degeneration, frailty syndrome-associated osteoporosis, and sarcopenia are major causes of physiological aging. Exercise interventions can be used as an effective strategy to reduce the risk of falls in the older population by improving their physical fitness and ameliorating physical degeneration [20]. The autonomy of the older population could be promoted by Pilates exercise in their daily living activities with a moderate level of certainty for the improvement of strength and falls [21]. In addition to strength and balance, Pilates could also bring the positive effects of postmenopausal BMD maintenance in multiple fracture risk factors [22].

Previous studies indicate that Pilates can effectively improve physical fitness in terms of aspects such as muscular strength, endurance, and flexibility, and that it can also have a disease mitigation effect, but the detailed Pilates exercise prescription and procedures were not sufficiently provided in previous studies. However, the effects of optimized Pilates exercise programs on functional fitness (body composition, cardiovascular endurance, agility, and dynamic/static balance) and metabolic rate should be further validated for community-dwelling populations. Accordingly, in the present study, we evaluated the effectiveness of a programed 12-week Pilates exercise intervention for improving the functional fitness, body composition, and basal metabolic rate of community-dwelling individuals, specifically middle-aged women. If shown to be effective, this evidence-based Pilates programs with a detailed exercise prescription (Table S1) could be a part of health promotion projects targeting aged populations for multiple benefits.

## 2. Materials and Methods

### 2.1. Experimental Design

We used a quasi-experimental design with a parallel control group for pretest–posttest evaluations following the 12-week Pilates exercise intervention. The detailed Pilates programs, including all the positions and numbers of repetitions, are presented in the Supplementary Data (Table S1). The assistant coach guided the participants through the correct Pilates posture and movements during the exercise intervention. A total of 50 healthy adult female volunteers who had not engaged in regular exercise in the previous three months were recruited as participants (Table 1). The participants with purposive sampling were allocated to the experimental (*n* = 25) or control (*n* = 25) group. During the experiment, five participants dropped out due to injuries, retirement, or family commitments. The study was reviewed and approved by the institutional review board of Saint Mary’s Hospital Luodong (Yilan, Taiwan; SMHIRB 102001), and the detailed experimental procedures were explained to the participants. All subjects provided informed consent before participating in the exercise program and assessments. The study was also publicly registered in clinicaltrials.gov (NCT05333484) on 19 April 2022. To eliminate possible confounding factors that may have affected the results, we applied the following exclusion criteria when recruiting participants: (1) cognitive function impairment, (2) spinal disease, (3) unsuitability for engaging in physical activity (e.g., because of severe cardiovascular disease), and (4) muscular strength affected by medication for nervous system diseases. All subjects participated in all assessments in the pretest and posttest sessions, which were conducted before and after the exercise intervention. The same physical fitness testers who did not know the participant allocation performed the physical fitness assessment as a single-blind strategy. The experimental group received 12 weeks of planned Pilates exercise training in two 60 min sessions per week, while the control group maintained their normal lifestyle. All the assessments and 12-week Pilates exercise interventions were acquired and performed in Camillians Saint Mary’s Hospital Luodong.

### 2.2. Primary Outcome—Body Composition

To assess the participants’ body composition, we measured their body weight, body mass index (BMI), body fat percentage, and muscular percentage in the upper and lower limbs. To obtain these measurements we conducted a multiple bioelectric impendent analysis (BIA) (Physion-XP or MD, Physion, Kyoto, Japan), which provides measurements that are strongly correlated with magnetic resonance imaging (MRI) measurements of 0.77–0.93 [23]. We conducted the BIA assessments under ambient temperature (20–30 °C) and relative humidity (30–85%). The source electrodes were placed on the dorsal surface of the third metacarpal bone of both hands and the third metatarsal bone of both feet. The detector electrodes were placed at the bilateral radial head, radial styloid process, fibular head, and lateral malleolus. The assessment was conducted using electric currents of 500 μA and 50 kHz. The BIA assessed fat percentage, muscle mass, limb muscle mass, and water percentage.

### 2.3. Primary Outcome—Basal Metabolic Rate

Indirect calorimeter assessments were conducted after a 3 h fast and at least 24 h of rest from moderate or intense physical activity. The handheld MedGem Indirect Calorimeter (HealtheTech, Golden, CO, USA), which measures oxygen consumption (assuming a respiratory quotient constant of 0.85), was used to measure basal metabolic rate, which it displays in cal/d, and volume of oxygen consumption (VO_2_) in mL/d. All measurements were conducted during the same time window (10:00–12:00). They required the creation of a leak-free seal between the participant’s mouth and a disposable mouthpiece with a nose clipper. Before the measurements were conducted, the participants were required to rest for 15 min to relax the body and then to sit in a reclining position in a comfortable chair during the measurement. The measurements were conducted until a 5 min steady-state period or a 10 min collecting period was recorded, whichever occurred first.

### 2.4. Primary Outcome—Functional Fitness

#### 2.4.1. Cardiovascular Capacity

The three-minute step test (TMST), a higher-stress test than the six-minute walk test (SMWT), was applied in the current study due to venue restrictions and participants’ fitness, and the significant correlation of TMST and SMWT was also demonstrated in a previous study [24]. The participants were required to stand on a 35 cm high wooden crate and perform 24 sets of consistent, repeated stepping up and down motions per minute for three minutes. One set comprised two stepping up and two stepping down motions performed within four beats. The preset rhythm for the test was 96 beats per minute, measured using an electronic metronome. The participants’ actual exercise time was recorded using a stopwatch. Regardless of whether they completed the full 3 min of the exercise or stopped midway, their recovery pulse rate during the periods 1 min–1 min 30 s, 2 min–2 min 30 s, and 3 min–3 min 30 s” or “1 min to 1 min 30 s, 2 min to 2 min 30 s, and 3 min to 3 min 30 s was measured with them in a sitting posture. The three pulse rates were input into the following formula to calculate the stepping cardiovascular function index: (exercise persistence duration × 100)/(2 × the sum of the three pulse rates).

#### 2.4.2. Core Strength

Muscular endurance was measured using the 1 min bent-knee sit-up test. The participants were asked to lie on their backs on a mat with their arms crossed over their chests and palms placed lightly on their shoulders. Their elbows were lifted off their chest, both knees were bent at 90 degrees, and the soles of their feet were placed flat firmly on the ground. They curled up to the point where both elbows touched the knees, followed by returning to the starting position (lying on the mat). The next sit-up could only be initiated after both shoulder blades had touched the mat. This set of sit-up motions was repeated for 60 s, and the number of sit-ups performed in this period was recorded as core strength endurance [25].

#### 2.4.3. Muscular Strength of Upper/Lower Limbs

We measured upper-extremity muscular strength via the grip strength test, with a grip strength meter. The participants were instructed as follows: find the best holding position (the ideal posture is to hold the handle tightly using the joints of the second segment of the fingers) and stand vertically with the hands hanging naturally at the side of the body, then hold the meter handle as tightly as possible without engaging in any other hand movements. They performed three sets of gripping motions, separated by a rest interval of at least 30 s, with each hand. The best result for each hand was recorded as the participant’s upper-extremity muscular strength [26].

We measured lower-extremity muscular strength via the 30 s chair stand test, using a straight-backed chair approximately 43 cm in height. The participants were required to sit on the chair with their back straight and their hands crossed over their chest, then perform repeated standing and sitting motions for 30 s. The number of repeats they performed within that time was recorded. It has been reported that the correlation coefficient between performance in this test and the single-repetition maximum leg press is 0.71–0.78 and the test–retest reliability is 0.86–0.92, which indicates that the 30 s chair–stand test exhibits good reliability and validity for testing lower-extremity muscular strength [26].

#### 2.4.4. Flexibility

We measured flexibility via the sit-and-reach test, using a triangular testing ruler. The participants sat on level ground or a mat with their legs separated, and the soles of their feet aligned with a measuring line. Their legs were straight, with the feet pointing upward. The participants then stretched forward as far as they could, with their hands overlapping, to push the point of the ruler with their fingertips. The distance (in cm) they could push it to without trembling and while maintaining a steady position for at least 2 s was recorded. A total of two trials were conducted, and the best (furthest) measurement was used as the participant’s flexibility score [26].

#### 2.4.5. Agility and Static/Dynamic Balance

We measured agility via the 8-foot timed up-and-go test using a stopwatch, measuring tape, obstacles, and a straight-backed chair approximately 43 cm in height. The participants were required to sit on the chair with their feet firmly placed on the floor and their hands on their thighs, with one leg placed slightly in front of the other. They were then timed while they stood and walked forward eight feet, bypassing the obstacles as they went, and then turned and walked back to the chair and sat down again. Two trials were conducted for each participant and the best (fastest) trial was used as their agility score [26].

We tested static balance using a single-leg stance with eyes closed test. The participants were required to place their hands on their hips and stand on level ground on one foot with their eyes closed. They were instructed to gently lean the lifted foot on the inner side of the ankle of the supporting foot. Timing (in seconds) was commenced immediately after the participant had stabilized themselves and the timer was stopped as soon as they set their foot down again [26].

We assessed dynamic balance using the functional reach test. Measurements were recorded using a measuring ruler attached to a wall, and the participants were requested to stand beside the wall and stretch their overlapped hands outward at 90 degrees to the body. They then bent over and stretched forward as far as they could without moving their legs or losing balance. The furthest distance (cm) they could reach with their fingertips was recorded. The average distance from two trials was used as their dynamic balance score [27].

### 2.5. Pilates Program Course Design

The 60 min Pilates sessions were held twice a week for 12 weeks. There were up to 25 participants in each session, and the sessions were conducted by a certified Pilates trainer. Each session included three stages: 10 min of warm-up exercises, 40 min of mat-based Pilates training, and 10 min of cool-down exercises. Each stage was adjusted according to the participants’ physical capacity. The content of the exercise program was determined according to their muscular strength, flexibility, and fitness levels. The training course was divided into three gradual stages: elementary, intermediate, and advanced. Each motion was performed in conjunction with a specific breathing pattern for effective muscle activations in each exercise. The full details of the Pilates exercise training course are shown in Table S1.

### 2.6. Sample Size Calculation

The equation of repeated measures design was applied to the sample size calculation by using G*power software. The parameters with an effect size of 0.5, a power of 80%, α error probability of 0.05, two group, and two times measurements, were estimated for a total of 42 participants (21 per group), an adequate sample size for the minimal requirement.

### 2.7. Statistical Analysis

We first analyzed the participants’ demographic variables using descriptive statistics. All dependent variables were then analyzed under the assumption of a normal distribution, using Kolmogorov–Smirnov tests for parametric (*p* > 0.05) and nonparametric (*p* < 0.05) analysis. Nonparametric (Wilcoxon signed-rank and Pearson chi-squared tests) and parametric (independent and paired *t*-tests) methods were used to evaluate the differences between and within groups by comparing pre- and posttest results. SPSS v. 22 (IBM, Armonk, NY, USA) was used for all statistical analyses, and statistical significance was assumed when the probability of a type I error was < 0.05.

## 3. Results

### 3.1. Participant Demographic Data

We recruited 50 middle-aged women within one month, and 45 of these participants completed the experimental interventions and assessments (22 and 23 in the experimental and control group, respectively). There were no significant differences between the two groups in terms of age, height, weight, marital status, educational attainment, or menopause status (*p* > 0.05; Table 1). The following primary outcomes were analyzed with 45 of these participants who completed the experimental interventions in the Experimental and Control groups. Both groups were followed up for adverse events for t months and were provided necessary medical assistance.

### 3.2. Effects of the Pilates Intervention on Functional Fitness

There were no significant differences in the results of any of the functional fitness tests between the groups before the intervention. In the posttest assessments, the experimental group performed significantly better than the control group in the 8-foot timed up-and-go (t(43) = 2.53, *p* = 0.015, d = 0.754, 95% C.I. = 0.149 to 1.359) and single-leg stance tests, but there were no significant differences between the groups in the other tests. There was a significant improvement in the sit-and-reach (t(21) = −2.507, *p* = 0.02, d = 0.534, 95% C.I. = −1.74 to −0.16), bent-knee sit-up (t(21) = −7.3, *p* < 0.0001, d = −1.55, 95% C.I. = −2.8 to −1.56), 30 s chair–stand (t(21) = −6.82, *p* < 0.0001, d = −1.45, 95% C.I. = −2.8 to −1.51), 8-foot timed up-and-go (t(21) = 2.36, *p* = 0.028, d = 0.501, 95% C.I. = 0.48 to 0.76), and functional reach (t(21) = −3.23, *p* = 0.004, d = −0.687, 95% C.I. = −2.14 to −0.466) tests within the experimental group but not in the control group (Table 2). The improvement in the posttest performance relative to the pretest assessments was significantly greater for the experimental group than the control group for the sit-and-reach (t(43) = −2.708, *p* = 0.010, d = 0.829, 95% C.I. = −2.12 to −0.31), bent-knee sit-up (t(43) = −6.44, *p* < 0.0001, d = 1.952, 95% C.I. = −2.75 to −1.43), 30 s chair–stand (t(43) = −4.4, *p* < 0.0001, d = 1.312, 95% C.I. = −2.92 to −1.08), 8-foot timed up-and-go (t(43) = 2.52, *p* = 0.016, d = 0.75, 95% C.I. = 0.11 to 1.02), and functional reach (t(43) = −3.124, *p* = 0.003, d = 0.93, 95% C.I. = −2.62 to −0.56) tests (Table 3). The clinical significance on fall incidence was validated by a significant difference from 0 to more than one fall on the related physical fitness (criteria), including the grip strength (1 kg), static balance (3 s), 8-foot timed up-and-go (1.7 s), and 30 s chair–stand (1.7 rep.) tests in a retrospective cross-sectional study [28]. According to the criterion of improved difference on fall incidence, the 30 s chair–stand, grip strength, static balance, 8-foot timed up-and-go tests in the experimental groups demonstrate a higher effectiveness of clinical difference than the control group.

### 3.3. Effects of the Pilates Intervention on Body Composition

The intervention significantly reduced BMI in the experimental group (t(21) = 4.61, *p* < 0.0001, d = 1.0, 95% C.I. = 0.214 to 0.567), while there were no changes in the control group (Table 4). Specifically, the within-group comparison also showed that the body fat percentage improved significantly in the experimental group after the intervention (t(21) = 4.73, *p* < 0.0001, d = 1.0, 95% C.I. = 0.476 to1.22), but that there were no significant changes in the control group.

### 3.4. Effects of the Pilates Intervention on Basal Metabolic Rate

The posttest basal metabolic rate of the experimental group was significantly higher than in the pretest (t(21) = −2.66, *p* = 0.015, d = 0.57, 95% C.I. = −209.9 to−25.7), and there were no significant changes in the control within the group (Table 5). In the posttest assessment, the basal metabolic rate of the experimental group was also significantly higher than the control between groups (t(43) = −2.72, *p* = 0.009, d = 0.811, 95% C.I. = −265.9 to −39.5). The pretest–posttest difference in the experimental group was also significantly greater than in the control group (t(43) = −2.45, *p* = 0.018, d = 0.73, 95% C.I. = −221.1 to −21.4).

## 4. Discussion

In the current study, middle-aged community-dwelling women participated in a 12-week Pilates program in an experiment we designed to evaluate the effects of the program on functional fitness, body composition, and basal metabolic rate. The experimental group exhibited significantly better improvements in body composition (BMI and body fat percentage), basal metabolic rate, and functional fitness (sit-and-reach, bent-knee sit-up, 30 s chair–stand test, 8-foot timed up-and-go, and functional reach) than the control group.

In terms of cardiorespiratory fitness, a recent meta-analysis showed that random effects meta-regressions based on baseline VO_2_ max were significant, which suggests that Pilates can improve cardiorespiratory fitness irrespective of health status [29]. An obese population also showed significantly higher cardiorespiratory VO_2_ max following Pilates interventions but did not significantly achieve the maximum heart rate with an internal training load [30]. In the current study, we assessed cardiovascular capacity based on heart rate recovery after a constant-pace step workout, which means the maximum heart rate could be an important limiting factor of Pilates exercise. We found no significant improvements in cardiovascular capacity, which is consistent with the study on the obese population [30]. Therefore, Pilates training may elevate cardiorespiratory fitness as a result of the training principle of controlled breathing, but different assessment methods may generate different results. The 6 min walk, a well-established metric for assessing aerobic capacity in elders, may be an alternative method for assessing cardiorespiratory capacity [31]. Another study showed that Pilates exercise training combined with inspiratory muscle training can significantly elevate pulmonary function and physical conditioning in the older population [32]. Cardiorespiratory VO_2_ max and the 6 min walk should be considered as alternative approaches to evaluating the effects of exercise interventions on aerobic capacity.

Patients with chronic nonspecific lower back pain receiving an eight-week Pilates intervention may show improvement in disability, pain, flexibility, and balance [33]. Both types of Pilates (mat and apparatus) can also improve lower- and upper-limb strength, aerobic endurance, lower- and upper-limb flexibility, and agility in older women [34]. In the current study, we observed significantly greater improvements in lower-limb strength, agility, flexibility, and dynamic balance following the Pilates intervention than in the control group, which is consistent with previous studies. Pilates interventions applied to different age populations may have different effects on static and dynamic balance. In a healthy volunteer university student cohort, an eight-week Pilates exercise program had beneficial effects on static balance, flexibility, abdominal muscle endurance, and abdominal and lumbar muscle activity, but not on dynamic balance [35]. Therefore, age-related atrophy in the cortical regions and corpus callosum may be associated with motor control deficits and motor decline, including balance deficits, coordination deficits, and slow movement [34]. The risk of falls in the older population may thus be further decreased through interdisciplinary collaboration and interventions, particularly focusing on exercise, improving medical conditions, and reducing environmental hazards [36]. In this context, the present Pilates program should be considered as an appropriate exercise prescription for the older female population. Our 12-week program may be beneficial for lower-limb strength, flexibility, agility, and balance in middle-aged women, which would help to reduce the risk of accidental falls.

Frailty is a multisystem aging syndrome and a clinical syndrome of increased vulnerability and functional impairment, accompanied by declining physiological and functional reserve and increasing health risks such as falls [37]. Frailty prevalence is significantly positively correlated with age, independent of assessment instrument, and ranged from 4–59% in community-dwelling older populations, with a higher prevalence in women than men [38]. With respect to the correlation between physical fitness and frailty, reduced lower-limb muscle strength is associated not only with signs of frailty, but also with reduced walking speed and hand grip strength [39]. Various exercise interventions, such as lower-limb resistance exercises, also result in a significant increase in cardiovascular endurance, lower-limb endurance and strength, and metabolic rate, although coordination and agility were not significantly improved, as indicated by the 8-foot timed up-and-go test [40]. Another training method, martial tai chi, also significantly improved balance and lower-limb strength in an older community-dwelling population [41]. In the Pilates intervention program in the current study, the participants’ performance in the 30 s chair–stand and 8-foot up-and-go tests, which assess lower-limb strength, coordination, and agility, improved significantly in the experimental group. Lower-limb strength may also be a key indicator of functional capacity in older adults and is also a crucial factor for determining whether older adults can live independently. The Pilates program in the current study may therefore be a potential strategy for improving the overall health of pre-frail and frail older people, especially their lower-limb strength, agility, and coordination.

In the current study, BMI and body fat percentage decreased significantly in the experimental group. This group’s basal metabolic rate also increased significantly following the Pilates training. With respect to the relationship between physical fitness and age-associated obesity, a cross-sectional study in northeast Brazil on four groups with varying levels of sarcopenia and obesity (sarcopenic obesity, 7.1%; obesity, 67.4%; sarcopenia, 12.4%; and normal, 13%) revealed that the prevalence of sarcopenic obesity was associated with poor physical performance [42]. In another study, middle-aged women underwent a 12-week Pilates program and assessments of body composition, hyperlipidemia indices, and lumbar muscle strength, revealing that Pilates exercise may be able to improve not only body composition, but also blood lipid levels, in middle-aged woman [43]. An eight-week Pilates program combined with a ball exercise program also improved weight, lean body mass, body fat percentage, and basal metabolic rate in sedentary obese women [44]. Another eight-week Pilates program appeared to mitigate the consequences of aging associated with an increased body fat percentage and reduced lean body mass in older women [45]. The 12-week Pilates exercise program in the current study may positively modulate body composition by elevating the basal metabolic rate and lower-limb strength, resulting in potential improved health and beneficial effects on weight management in older people. The relation between physical activity level and functional fitness with age-related decline was elucidated with a comparison of 60–69-years-old and 70–80-years-old populations [46]. Physical fitness demonstrates a decreasing trend with ageing if the appropriate exercise is not implemented. In the comparison of adult (21–60-years-old) and geriatric (>60-years-old) groups, one study also revealed that age and weight are factors that influence physical activity levels [47]. Thus, the significant difference decrease in physical fitness was not observed in the Control group, possibly because the duration was only 12 weeks (short-duration observation). The results from the differences in physical fitness in the Control group also reinforce the effectiveness of Pilates implementation on the older female population compared to the Experimental group.

Upper-limb strength and cardiovascular endurance did not appear to improve as a result of the current exercise program. Pilates props and accessories, such as balls, elastic bands, foam cushions, and fitness circles, may also be integrated into an exercise program in a future study to test for their effects on various aspects of physical fitness. Furthermore, cardiorespiratory and cardiovascular capacity findings may be further validated by improving the experimental design for assessing older populations, and the different interference factors, such as health conditions, gender, age, lifestyles, etc., could also contribute the different effects with exercise training. Finally, it may be advisable to further combine the Pilates program with nutritional supplementation and dietary management to achieve potential physiological and fitness benefits and promote health and ameliorate disease risk in older populations. The current study also demonstrates the basic Pilates exercise prescription on the middle-aged women population for the reference of instructors on health promotion projects. However, the effects of programed Pilates on the older population with older age, gender, and other chronic disease should be further validated for potential health benefits. Furthermore, the establishment of a data base related to age, gender, demographic variables, disease history, health condition, physical activities, and body composition in the elderly population could be important information to objectively evaluate the effects of public policy implementation, such as an exercise program and nutritional recommendations, in the future.

## 5. Conclusions

The 12-week Pilates exercise program (Table S1) is practical to implement in communities to promote health and improve body composition, functional fitness, and basal metabolic rate, which may help to prevent age-associated frailty and reduce the risk of accidental falls in middle-aged women populations.

## Figures and Tables

**Table 1:** Participant demographic data.

	Total (*n* = 45)		Experimental Group (*n* = 22)		Control Group (*n* = 23)		*p*
Age (years)	55.13 ± 5.49		54.64 ± 5.23		55.61 ± 5.80		0.488
Height (cm)	155.18 ± 4.74		155.61 ± 4.97		154.76 ± 4.59		0.453
Weight (kg)	57.16 ± 10.01		58.44 ± 9.27		55.93 ± 10.75		0.216
	*n*	(%)	*n*	(%)	*n*	(%)	
Education							0.823
Elementary	4	8.9	1	4.5	3	13.0	
Junior	7	15.6	3	13.6	4	17.4	
Senior	16	35.6	8	36.4	8	34.8	
College	15	33.3	8	36.4	7	30.4	
Graduate	3	6.7	2	9.1	1	4.3	
Marital status							0.230
Single	3	6.7	2	9.1	1	4.3	
Married	38	84.4	19	86.4	19	82.6	
Divorced	1	2.2	1	4.5	0	0.0	
Widowed	3	6.7	0	0	3	13.0	
Menopause							0.776
No	9	20.0	4	18.2	5	21.7	
Yes	36	80.0	18	81.8	18	78.3	

**Table 2:** Effects of 12-week Pilates program on functional fitness.

Test		Pretest	Posttest	Within *p*
		Mean ± SD	Mean ± SD	
3 min step	Exp.	55.42 ± 5.76	55.37 ± 5.32	0.651
	Con.	55.40 ± 5.35	55.61 ± 5.54	0.407
	Between	*p* = 0.992	*p* = 0.440	
Sit-and-reach (cm)	Exp.	27.00 ± 9.04	27.95 ± 8.83 ^#^	0.001
	Con.	25.65 ± 9.58	25.39 ± 9.44	0.527
	Between	*p* = 0.630	*p* = 0.353	
Bent-knee sit-up (rep.)	Exp.	6.91 ± 8.47	9.09 ± 8.40 ^#^	0.0001
	Con.	6.78 ± 7.16	6.87 ± 6.99	0.527
	Between	*p* = 1.00	*p* = 0.316	
Grip strength (kg)	Exp.	20.05 ± 3.07	20.17 ± 3.12	0.337
	Con.	21.21 ± 3.55	20.98 ± 3.31	0.781
	Between	*p* = 0.261	*p* = 0.399	
30 s chair–stand (rep.)	Exp.	16.05 ± 4.33	18.22 ± 4.42 ^#^	0.0001
	Con.	16.09 ± 3.08	16.26 ± 3.49	0.508
	Between	*p* = 0.971	*p* = 0.182	
8-foot timed up-and-go (s)	Exp.	5.56 ± 0.62	5.15 ± 0.72 *^,#^	0.028
	Con.	5.66 ± 0.85	5.83 ± 1.02 ^*^	0.258
	Between	*p* = 0.644	*p* = 0.015	
Single-leg stance with eyes closed (s)	Exp.	8.12 ± 13.13	8.72 ± 12.4 *	0.086
	Con.	4.00 ± 3.65	3.94 ± 3.08	0.867
	Between	*p* = 0.251	*p* = 0.047	
Functional reach (cm)	Exp.	30.9 ± 4.38	32.2 ± 4.55 ^#^	0.004
	Con.	31.9 ± 3.05	31.6 ± 3.23	0.375
	Between	*p* = 0.369	*p* = 0.627	

Con., control group (*n* = 23); Exp., experimental group (*n* = 22); rep., number of complete repeats performed; * *p* < 0.05 (between groups) and ^#^ *p* < 0.05 (within groups).

**Table 3:** The clinical significance of fall prevention and within-group effects with 12-week Pilates program (pre- vs. posttest comparisons).

Test	Change within Experimental Group	Change within Control Group	*p*	Clinical Significance (Experimental vs. Control Groups) (%)
	Mean ± SD	Mean ± SD		
3 min step	−0.04 ± 2.26	0.20 ± 3.94	0.351	-
Sit-and-reach (cm)	0.95 ± 1.78 *	−0.26 ± 1.17	0.012	-
Bent-knee sit-up (rep.)	2.18 ± 1.40 *	0.09 ± 0.66	0.0001	-
Grip strength (kg)	0.12 ± 0.68	−0.22 ± 1.21	0.413	9.1 vs. 0
30 s chair–stand (rep.)	2.18 ± 1.5 *	0.17 ± 1.55	0.000	68.2 vs. 30.4
8-foot timed up-and-go (s)	−0.40 ± 0.81 *	0.16 ± 0.70	0.025	4.5 vs. 0
Single-leg stance with eyes closed (s)	0.59 ± 1.55	−0.05 ± 0.95	0.069	9.1 vs. 0
Functional reach (cm)	1.31 ± 1.89 *	−0.28 ± 1.51	0.001	-

Con., control group (*n* = 23); Exp., experimental group (*n* = 22); rep., number of complete repeats performed. *, represents the significant difference between groups (*p* < 0.05). The effectiveness of clinical significance (%) in elderly fall prevention was evaluated by the within-group difference according to the difference criterion of fall incidence in indicated physical fitness.

**Table 4:** Effects of 12-week Pilates on body composition.

		Pretest	Posttest	*p*
		Mean ± SD	Mean ± SD	
Weight (kg)	Exp.	58.44 ± 9.27	58.35 ± 9.17	0.906
	Con.	55.93 ± 10.7	56.23 ± 10.6	0.079
BMI (kg/m^2^)	Exp.	24.48 ± 2.80	24.09 ± 2.60 **^#^**	0.020
	Con.	23.26 ± 3.89	23.33 ± 3.78	0.278
Body fat percentage (%)	Exp.	27.72 ± 3.97 *	26.87 ± 4.74 *^,#^	0.0001
	Con.	24.70 ± 4.83	24.80 ± 4.74	0.109
Lean body mass percentage (Upper limbs) (%)	Exp.	2.98 ± 0.42	3.16 ± 0.44	0.224
	Con.	3.19 ± 0.72	3.16 ± 0.71	0.927
Lean body mass percentage (Lower limbs) (%)	Exp.	16.34 ± 2.38	19.82 ± 4.32	0.073
	Con.	17.82 ± 2.51	18.25 ± 3.15	0.820

BMI, body mass index; Con., control group (*n* = 23); Exp., experimental group (*n* = 22); The symbols (* and **^#^**) represent the significant difference between and within groups, respectively (*p* < 0.05).

**Table 5:** Effects of 12-week Pilates on basal metabolic rate.

		**Pretest**	**Posttest**	***p***
		**Mean ± SD**	**Mean ± SD**	
BMR (kcal/day)	Exp.	1104.1 ± 159	1221.9 ± 193 *^,#^	0.015
	Con.	1072.6 ± 165	1069.2 ± 184	0.726
(kcal/h/kg)	Exp.	19.21 ± 3.19	21.26 ± 3.57	0.007
	Con.	23.26 ± 3.89	23.33 ± 3.78	0.879
		**Change within Group**		
		**Mean ± SD**		
BMR (kcal/day) (kcal/h/kg)	Exp.	117.9 ± 207 *		0.018 0.021
		(2.05 ± 3.58) *		
	Con.	–3.39 ± 112		
		(–0.13 ± 2.25)		

BMR, basal metabolic rate; Con., control group (*n* = 23); Exp., experimental group (*n* = 22). The symbols (* and ^#^) represent the significant difference between and within groups, respectively (*p* < 0.05).

## Data Availability

The datasets generated and analyzed during the current study are not publicly available but are available from the corresponding author who was an organizer of the study.

## Supplementary Materials

The following supporting information can be downloaded at: https://www.mdpi.com/article/10.3390/ijerph192316157/s1, Table S1: The programed 12-week Pilates exercise designs.
