# Supplementary material for: Effects of a 12-Week Pilates Program on Functional Physical Fitness and Basal Metabolic Rate in Community-Dwelling Middle-Aged Women: A Quasi-Experimental Study
Source: Int J Environ Res Public Health. 2022 Dec 2;19(23):16157. doi: 10.3390/ijerph192316157 (PMC9737173; doi:10.3390/ijerph192316157)
Supplement: Supplementary file 1 [file ijerph-19-16157-s001.zip › ijerph-2062542-supplementary.pdf]

Supplementary data

Table S1. The programed 12-week Pilates exercise designs

Exercise/postures	Duration/Repetitions	Weeks
**Warming up**	**10 minutes**	1-12
Breathing	4 minutes	1-12
Pelvic clock	8 repetitions	1-12
Arm circles	8 repetitions	1-12
Scapula isolation	8 repetitions	1-12
Basic bridging	8 repetitions	1-12
**Main Exercise**	**40 minutes**	
Pelvic curls	10 repetitions	1-6
Knee fold	10 repetitions	1-6
Chest lift	10 repetitions	1-6
Oblique reaches	10 repetitions	1-6
Knee side to side	10 repetitions	1-6
The hundred	10 repetitions	1-12
Side leg lifts	10 repetitions	1-6
Roll up	10 repetitions	1-6
Leg circles	10 repetitions	1-6
Double leg stretch	10 repetitions	1-6
Single leg stretch	10 repetitions	7-12
Crisscross	10 repetitions	7-10
Saw	8 repetitions	7-12
Spine twist	8 repetitions	7-12
Roll like a ball	8 repetitions	7-12
Roll over	8 repetitions	7-12
Shoulder bridge	8 repetitions	7-12
Side kick	8 repetitions	7-10
Side leg kick	8 repetitions	7-10
Double leg kick	8 repetitions	7-10
Swimming	8 repetitions	7-12
Neck pull	8 repetitions	10-12
Leg pull down	8 repetitions	10-12
Side kick kneeling	8 repetitions	10-12
Hip circle	8 repetitions	10-12
Bend	8 repetitions	10-12
**Cooling down**	**10 minutes**	

Upper back stretch	8 repetitions	1-12
Cat stretch	8 repetitions	1-12
Spine stretch	8 repetitions	1-12
Rest position	4 minutes	1-12
